# Epithelioid granuloma mimicking lung cancer showed intense uptake on [^18^F]FAPI-74 PET

**DOI:** 10.1007/s00259-023-06478-9

**Published:** 2023-10-28

**Authors:** Tadashi Watabe, Ivan Ray David, Toru Kimura, Takashi Hiroshima, Mitsuaki Tatsumi, Sadahiro Naka, Takashi Kamiya, Eriko Fukui, Takashi Kanou, Naoko Ose, Soichiro Funaki, Yuriko Mori, Jens Cardinale, Hiroki Kato, Eiichi Morii, Yasushi Shintani, Frederik L. Giesel

**Affiliations:** 1 Department of Nuclear Medicine and Tracer Kinetics, Graduate School of Medicine, Osaka University, 2-2 Yamadaoka, Suita, Osaka 565-0871 Japan; 2 Institute for Radiation Sciences, Osaka University, Toyonaka, Japan; 3 Department of Nuclear Medicine, Jose R. Reyes Memorial Medical Center, Manila, Philippines; 4 Department of General Thoracic Surgery, Graduate School of Medicine, Osaka University, Suita, Japan; 5 Department of Radiology, Osaka University Hospital, Suita, Japan; 6 Department of Pharmacy, Osaka University Hospital, Suita, Japan; 7 Department of Nuclear Medicine, University Hospital Duesseldorf, Medical Faculty, Heinrich-Heine-University, Duesseldorf, Germany; 8 Department of Pathology, Graduate School of Medicine, Osaka University, Suita, Japan


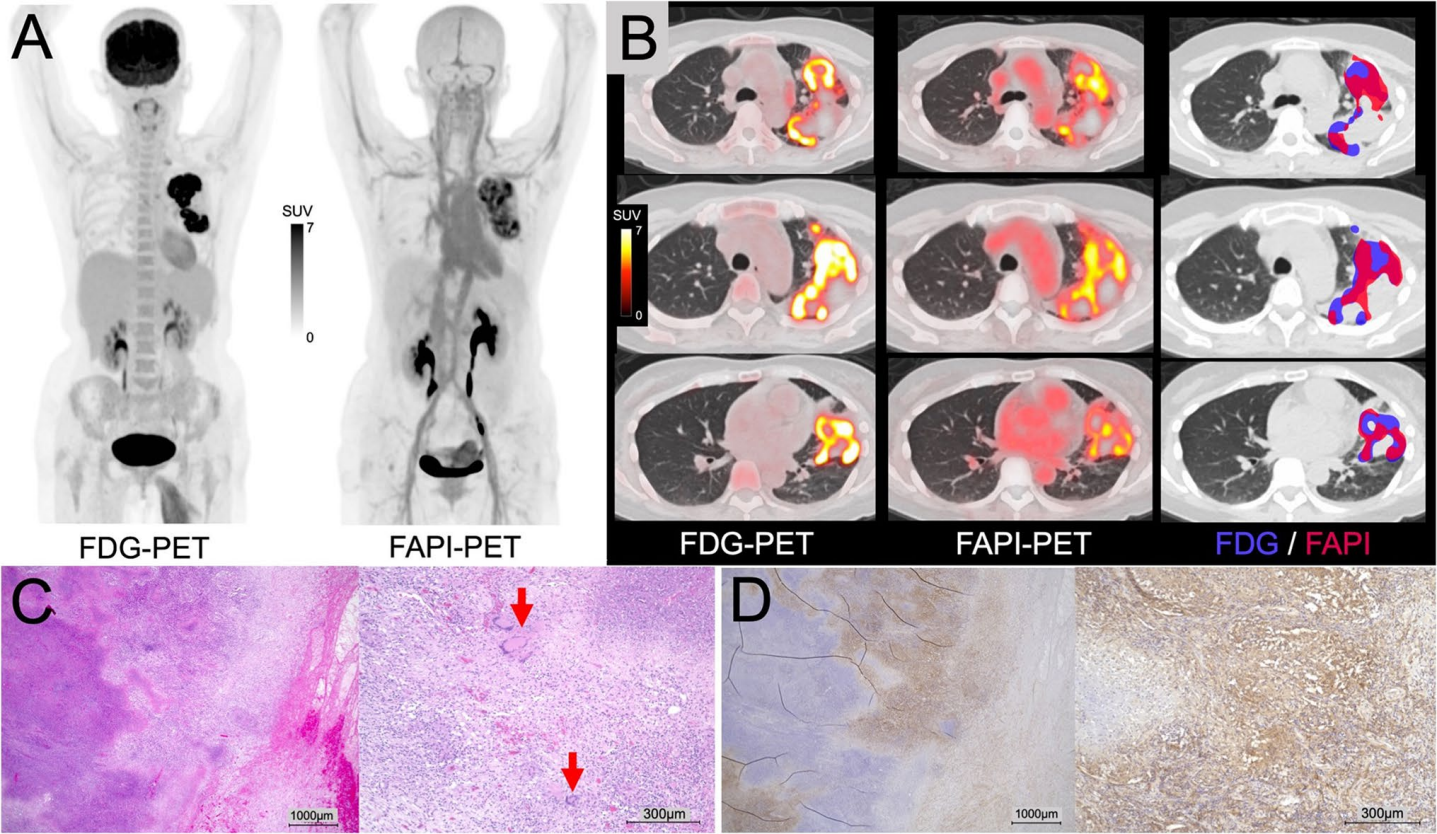


A 54-year-old woman suspected of lung cancer underwent [^18^F]FDG and [^18^F]FAPI-74 PET scans. CT revealed a 7-cm lesion in the left upper lobe. Despite detecting only necrotic cells on biopsy during bronchoscopy, malignancy was strongly suspected, leading to a planned lobectomy.

[^18^F]FDG PET-CT showed a highly avid mass (SUVmax: 14.2) in the left upper lung lobe, along with suspicious lymph node metastases with mild uptake in the left hilar, thoracic para-aortic, and left supraclavicular regions (A: MIP; B: PET/CT fusion, left). Similarly, [^18^F]FAPI-74 PET-CT also showed intense uptake (SUVmax: 9.02) with heterogeneous distribution in the same lesion, but with slightly larger extent (A and B, right), and no significant uptake was observed in the lymph nodes. Some areas displayed faint to mild FAPI uptake mismatched with FDG.

Histopathology revealed epithelioid granuloma (C: arrows indicate Langhans giant cells in H&E staining), but results for Grocott stain, PAS stain, and Ziehl–Neelsen stain were all negative. Immunohistochemical staining for FAP showed some areas with FAP expression surrounding the necrotic tissue in heterogenous uptake on [^18^F]FAPI-74 PET (D).

FAPI, an emerging pan-tumour tracer, also accumulates in chronic infections, leading to false positives [1]. Literature supports FAPI PET/CT’s use in granulomatous conditions like tuberculosis [2–4]. The mismatch between FAPI and FDG accumulation may represent varying lesion stages. The FDG-avid lesion might represent an inflammatory-proliferative stage, while the FAPI-positive lesion could represent a fibrotic phase [5]. Evaluation of FAPI uptake in non-malignant tumours will expand the clinical utility of FAPI-PET across diverse scenarios.
